# XAP5 CIRCADIAN TIMEKEEPER Affects Both DNA Damage Responses and Immune Signaling in *Arabidopsis*

**DOI:** 10.3389/fpls.2021.707923

**Published:** 2021-10-01

**Authors:** Roderick W. Kumimoto, Cory T. Ellison, Tania Y. Toruño, Aurélie Bak, Hongtao Zhang, Clare L. Casteel, Gitta Coaker, Stacey L. Harmer

**Affiliations:** ^1^Department of Plant Biology, University of California, Davis, Davis, CA, United States; ^2^Department of Plant Pathology, University of California, Davis, Davis, CA, United States; ^3^Department of Plant Pathology and Plant-Microbe Biology, Cornell University, Ithaca, NY, United States

**Keywords:** immune signaling, DNA damage response, circadian, phase, *Pseudomonas syringae*

## Abstract

Numerous links have been reported between immune response and DNA damage repair pathways in both plants and animals but the precise nature of the relationship between these fundamental processes is not entirely clear. Here, we report that XAP5 CIRCADIAN TIMEKEEPER (XCT), a protein highly conserved across eukaryotes, acts as a negative regulator of immunity in *Arabidopsis thaliana* and plays a positive role in responses to DNA damaging radiation. We find *xct* mutants have enhanced resistance to infection by a virulent bacterial pathogen, *Pseudomonas syringae* pv. *tomato* DC3000, and are hyper-responsive to the defense-activating hormone salicylic acid (SA) when compared to wild-type. Unlike most mutants with constitutive effector-triggered immunity (ETI), *xct* plants do not have increased levels of SA and retain enhanced immunity at elevated temperatures. Genetic analysis indicates *XCT* acts independently of *NONEXPRESSOR OF PATHOGENESIS RELATED GENES1* (*NPR1*), which encodes a known SA receptor. Since DNA damage has been reported to potentiate immune responses, we next investigated the DNA damage response in our mutants. We found *xct* seedlings to be hypersensitive to UV-C and γ radiation and deficient in phosphorylation of the histone variant H2A.X, one of the earliest known responses to DNA damage. These data demonstrate that loss of *XCT* causes a defect in an early step of the DNA damage response pathway. Together, our data suggest that alterations in DNA damage response pathways may underlie the enhanced immunity seen in *xct* mutants.

## Introduction

The genome sequencing of a wide range of organisms has enabled the identification of genes that are widely conserved and yet generally maintained in a single copy per genome. Such genes are likely under stringent selection for retention and strong dosage constraint, and are more likely to perform essential functions than genes present in multiple copies per genome (Waterhouse et al., 2011). One such gene is *XAP5 CIRCADIAN TIMEKEEPER* (*XCT*), which we originally identified on the basis of the short-period circadian phenotype of *Arabidopsis thaliana* plants mutant for this gene (Martin-Tryon and Harmer, 2008). Highly conserved XCT homologs are present in most eukaryotes and have been shown to be nuclear localized in *Arabidopsis*, fission yeast, and *Chlamydomonas* (Matsuyama et al., 2006; Martin-Tryon and Harmer, 2008; Li et al., 2018). In most organisms, these homologs are termed *XAP5* genes, named for the highly conserved domain of unknown function found in the C-termini of the encoded proteins.

XAP5 CIRCADIAN TIMEKEEPER homologs have been reported to act in distinct biological processes in different organisms. In *Arabidopsis*, XCT plays roles in regulation of the circadian clock, light responses, gene expression, ethylene signal transduction, and biogenesis of small RNAs (Martin-Tryon and Harmer, 2008; Ellison et al., 2011; Fang et al., 2015; Xu et al., 2017). In the ciliate *Chlamydomonas reinhardtii*, the XCT homolog XAP5 has been reported to act as a transcription factor to control flagellar assembly (Li et al., 2018). In the nematode *Caenorhabditis elegans*, downregulation of the XCT homolog causes embryo lethality (Piano et al., 2002). Finally, we have previously shown that deletion of the *xap5* locus in the fission yeast *Schizosaccharomyces pombe* negatively affects growth and that this phenotype is rescued by *Arabidopsis XCT* (Anver et al., 2014). Subsequent genetic and biochemical analyses in this yeast revealed that Xap5 is a chromatin-associated protein affecting chromatin regulation in a manner similar to the variant histone H2A.Z.

Chromatin dynamics are key to a wide range of nuclear processes, including regulation of gene expression, silencing of heterochromatin, and repair of DNA damage (Vergara and Gutierrez, 2017). For example, in plants as well as in animals, chromatin remodeling complexes and histone variants play important roles in the maintenance of genome stability after diverse types of DNA damage (Talbert and Henikoff, 2014; Han et al., 2015; Gursoy-Yuzugullu et al., 2016). Modification of chromatin structure is especially important in the repair of DNA double-strand breaks, a very cytotoxic form of DNA damage (Kinner et al., 2008). A conserved eukaryotic response to stresses that cause double-strand breaks is the activation of two related kinases, ATAXIA TELANGIECTASIA MUTATED (ATM) and ATM AND RAD3-RELATED (ATR), followed by their rapid phosphorylation of thousands of molecules of the variant histone H2A.X near the sites of DNA damage to generate a modified form termed γ-H2A.X (Rogakou et al., 1999). γ-H2A.X then acts as a platform for the recruitment and retention of DNA damage repair factors (Van and Santos, 2018). In plants, activation of ATM also leads to the phosphorylation and activation of SUPPRESSOR OF GAMMA RESPONSE 1 (SOG1; Yoshiyama et al., 2013, 2017), a transcription factor that induces expression of genes involved in DNA repair (Yoshiyama et al., 2009).

There is evidence that damaged DNA can induce pathogen defense pathways. In animals, pathogen-derived DNA and damaged host DNA can both induce immune signaling (Motwani et al., 2019). The role of DNA damage in plant defense signaling is less clear (Camborde et al., 2019). Plants use two main types of immune receptors to defend against pathogens. Basal defense pathways are activated upon the sensing of microbial features by extracellular receptors, inducing pattern-triggered immunity (PTI). However, successful pathogens deliver effector molecules into plant cells to inhibit defense, enable nutrient acquisition, and reprogram their hosts. In response, plants have evolved intracellular immune receptors that directly or indirectly recognize these effectors (Jones et al., 2016). Direct or indirect recognition of pathogen effectors triggers local effector-triggered immunity (ETI), which can induce systemic acquired immunity to afford disease resistance throughout the organism.

Activation of both PTI and ETI are associated with the synthesis of the defense hormone salicylic acid (SA; Liu et al., 2020). An SA receptor, *NONEXPRESSOR OF PATHOGENESIS RELATED GENES1* (*NPR1*), is an important mediator of long-lasting, broad-spectrum defense responses (Wu et al., 2012; Manohar et al., 2014; Ding et al., 2018). Many mutants with enhanced basal pathogen resistance have alterations in ETI signaling components. This enhanced basal immunity is usually both characterized by elevated levels of SA (Durner et al., 1997; Petersen et al., 2000; Li et al., 2001; Huang et al., 2010; Zhang et al., 2010) and suppressed at higher temperatures (Yang and Hua, 2004; Wang et al., 2009; Kim et al., 2010; Zhu et al., 2010; Alcazar and Parker, 2011).

The precise nature of the relationship between DNA damage response pathways and plant immunity is currently unclear (Yan et al., 2013; Song and Bent, 2014; Rodriguez et al., 2018). Some have reported that DNA damage promotes plant immunity by activating pathways that function in parallel to the ETI pathway described above *via* SA-independent mechanisms (Li et al., 1999; Durrant et al., 2007; Wang et al., 2010; Song et al., 2011; Yan et al., 2013; Ogita et al., 2018). Consistent with this view, the SOG1 transcription factor directly controls expression of genes involved in plant defense signaling in addition to promoting expression of genes involved in the DNA damage response (Yoshiyama et al., 2013; Ogita et al., 2018). Others have suggested that the DNA damage observed during pathogen infection is simply a consequence of the programmed cell death that occurs during ETI (Rodriguez et al., 2018). It has been proposed that these conflicting views could be reconciled if DNA damage and the activation of repair pathways play multiple roles during plant immune responses (Camborde et al., 2019). Clearly, further work is needed to resolve the nature of the links between plant immune signaling and DNA damage response pathways.

In this study, we demonstrate that *Arabidopsis XCT* negatively regulates defense pathways independently of the SA receptor NPR1. Unlike typical mutants with constitutive ETI, disease resistance is not temperature sensitive, SA levels are not elevated, and no spontaneous lesions are observed in *xct* mutants. Transcriptome analysis of *xct* mutants reveals significant overlap of genes upregulated in these plants and those induced in response to SA and to DNA-damaging treatments. Consistent with a role for XCT in DNA damage response pathways, we demonstrate that *xct* mutants are hypersensitive to DNA damaging agents. This putative role for XCT in DNA damage response signaling may be fairly direct, as *xct* mutants have very severe defects in phosphorylation of H2A.X after treatment with ionizing radiation. Thus our data demonstrate that XCT is a negative regulator of plant immunity and suggest this may be related to its role in DNA damage response pathways.

## Materials and Methods

### Plant Materials

The *xct-1*, *xct-2*, *xct-2 XCT::XCT-YFP-HA*, *xct-5*, *npr1-1*, *arp6-1*, and *sog1-1* genotypes have been previously described (Cao et al., 1994; Preuss and Britt, 2003 #1297; Deal et al., 2005; Martin-Tryon and Harmer, 2008; Xu et al., 2017).

### Growth Conditions

Seedlings were germinated and grown on plates for the circadian, hypocotyl, chlorophyll content, genotoxic agent, and gene expression assays. Seeds were plated on media containing 1x Murashige and Skoog (Research Products International Corp.), 0.7% agar (EMD Chemicals Inc.), and sucrose [Fisher Chemical; 3% sucrose for circadian, hypocotyl, and chlorophyll content assays; 1.5% sucrose for RNA-seq and quantitative PCR (qPCR) analysis]. Unless otherwise specified, seedlings were grown in 12h light/12h dark (12:12L/D) cycles under 55μmol m^−2^ s^−1^ white light at constant 22°C.

### RNA-Seq Analysis

Plant tissue for transcriptome analysis was obtained as follows. Seeds of the genotypes Col, *xct-1*, and *xct-2* were sterilized, cold stratified, and sown onto 1X MS media containing 1.5% (w/v) sucrose. Following cold stratification, seed plates were transferred to 12h light/12h dark (12:12 L/D) cycles under 55μmol m^−2^ s^−1^ white light at constant 22°C. Plants were grown in this condition for 11days and on the 12th day of growth at 3h after dawn plant tissue from each genotype was harvested and flash frozen in liquid nitrogen. For RNA-seq library construction, total RNA was isolated from frozen plants using the TRIzol reagent and protocol (Invitrogen) according to manufacturer instructions. The Ribo-Zero rRNA Removal Kit-Plant Leaf (Epicentre) was used according to manufacturer instructions to remove rRNAs from the total RNA population. Around 100ng of RNA from the rRNA-depleted total RNA samples were then used to construct strand-specific libraries for RNA sequencing as previously described (Wang et al., 2011).

Nine libraries (three each generated from Col, *xct-1*, and *xct-2*) were multiplexed and sequenced on two lanes of an Illumina HiSeq. About 100cycle runs were used to generate single-end reads, which were quality filtered such that only those with a quality cut-off score of 20 or greater over 85% or more of the 100-nucleotide sequence were retained. Reads were separated by their barcodes and Illumina adapter contamination was removed, resulting in a total of 58.0, 53.3, and 48.7 million high-quality reads for the Col, *xct-1*, and *xct-2*, respectively. Reads were mapped to *Arabidopsis* TAIR10 representative gene models with BWA (Li and Durbin, 2009) using the parameters –k 1 –l 25 –n 0.1 –e 12 –i 100. About 80.1% of the Col, 80.1% of the *xct-1*, and 77.2% of the *xct-2* reads mapped to the TAIR10 gene models.

To identify genes differentially expressed in the mutants relative to the wild-type control samples, we used the Bioconductor package edgeR (Robinson et al., 2010; version 3.22.3). This package uses an empirical Bayesian approach based upon the negative binomial distribution to model digital expression data. Before comparing gene expression between genotypes, we first removed all genes encoded by the mitochondrial and chloroplast genomes to help control for the delayed chloroplast maturation phenotype of *xct-2* (Martin-Tryon and Harmer, 2008). We imposed an expression value threshold, excluding genes that did not have at least one read per million reads in at least three libraries. Genes differentially expressed between genotypes were determined using the exact test for the negative binomial distribution using approach of Benjamini and Hochberg (1995) to control the false discovery rate (FDR) to below 5%. Differentially expressed genes were compared to those previously identified as regulated by the circadian clock (Hsu and Harmer, 2012) as described in the text.

Enrichment of differentially assessed genes with known molecular functions in the RNA-seq dataset was assessed using the clusterProfiler R package (v 3.8.1; Yu et al., 2012) with FDR<0.01 using BH correction. Overlap between genes differentially expressed in *xct* mutants and in previously published studies was visualized using the Vennerable R package (Swinton, 2019) and significance assessed using Fisher’s exact test.

### Phenotypic Assays

For bacterial growth assays, *Arabidopsis* seeds were sown on soil and stratified for 2days in the dark at 4°C. For the experiments conducted at lower temperatures, *Arabidopsis* plants were grown in a controlled environment chamber at 23°C, 70% relative humidity, light intensity of 100μmol m^−2^ s^−1^, and a 10/14h light/dark photoperiod. For the higher temperature experiments, *Arabidopsis* plants were grown in a controlled environment chamber at 28°C, 70% relative humidity, light intensity of 120μmol m^−2^ s^−1^, and an 11/13h light/dark photoperiod. *Pseudomonas syringae* pv. *tomato* DC3000 was grown at 28°C on NYGA media supplemented with 100mg ml^−1^ rifampicin and 25mg ml^−1^ kanamycin. Bacterial growth assay was performed as described previously (Kim et al., 2005). Bacterial cells at a concentration of 1×10^5^ colony forming units (CFU) ml^−1^ were infiltrated into leaves of 4-week-old *Arabidopsis* plants. Bacterial titers were measured 0 and 3days post inoculation. The experiments were repeated three times with similar results. For salicylic acid quantification, six plates of seedlings, grown for 9days in light/dark cycles, were used for each treatment. From each plate, 100mg of plants were collected and pooled separately, flash frozen in liquid nitrogen and pulverized. Hormones were extracted from frozen tissue as previously described (Casteel et al., 2015). From each sample 10μl was injected into A Zorbax Extend-C18 column 3.0x150mm (Agilent) using a 6,420 triple quad liquid chromatography-tandem mass spectrometry machine (Agilent).

The circadian period analysis was performed as previously described (Hsu et al., 2013) with minor alterations. Seedlings were entrained in 12h white light (50–60 μmol m^−2^ s^−1^; cool white fluorescent bulbs):12h dark at 22°C for 6days before being released to constant monochromatic red plus monochromatic blue light (35 μmol m^−2^ s^−1^ red light, 35 μmol m^−2^ s^−1^ blue light; XtremeLux, Santa Clara, CA, United States). Luciferase activity was monitored using a cooled CCD camera (DU434-BV, Andor Technology) and free-running period estimated by fast Fourier transformed nonlinear least squares (Plautz et al., 1997).

UV-C treatments were performed as previously described (Rosa et al., 2013) with minor alterations. About 4-day-old seedlings were treated with the indicated doses of UV-C irradiation using a Stratalinker, model 1800 (Stratagene). Plants were then returned to the growth chamber for an additional 8days and then visually assessed for emergence of true leaves. Gamma irradiations were performed as previously described (Johnson et al., 2018) with minor alterations. Seedlings were grown on vertically oriented plates in 16h light/8h dark cycles under 80μmol m^−2^ s^−1^ white light at constant 22°C. Starting on day 4 plates were scanned daily using an Epson Perfection V800 scanner. On day 5, seedlings were γ-irradiated with 150Gy at a dose rate of 1.8Gy/min. Total root length was measured every day from the scanned images using ImageJ (ver. 1.43; Schneider et al., 2012). Relative growth rate of primary roots was calculated using previously described methods (Hoffmann and Poorter, 2002). Statistical significance for response to genotoxic agents was assessed using the R language and environment and the lme4 and lmerTest R packages (Bates et al., 2015; Kuznetsova et al., 2017; R Core Team, 2018).

### Quantitative Reverse-Transcriptase PCR

Seedlings were grown on plates and either sprayed with 0.3mM salicylic acid (Van Leeuwen et al., 2007) or mock treated 3h after dawn on the 9th day after germination. Tissue was collected 24h post treatment. Total RNA extraction was carried out using Trizol reagent (Life Technologies). Total RNA was then DNase treated using the Qiagen RNase free DNase kit protocol (Qiagen). RNA was quantified using a Nanodrop and cDNA synthesis carried out using Super Script III Reverse Transcriptase as specified by the manufacturer’s protocol (ThermoFisher). Quantitative reverse-transcriptase PCR (qRT-PCR) was performed as previously described (Shalit-Kaneh et al., 2018) using a BioRad CFX96 thermocycler (Bio-Rad). Primers for qRT-PCR are listed in Supplementary Table 3. Cq values and StdE were obtained from the BioRad CFX96 software package. Statistical significance was assessed by linear mixed-effect models with treatment and genotype as fixed effects and trial as a random effect using the lme4 and lmerTest R packages (Bates et al., 2015; Kuznetsova et al., 2017; R Core Team, 2018).

### γ-H2A.X Immunoblotting

Gamma irradiations were performed as previously described (Friesner et al., 2005) with minor alterations. Seedlings were grown on plates in 12h light/12h dark cycles under 55μmol m^−2^ s^−1^ white light at constant 22°C. On day 11, seedlings were irradiated with 50Gy using a ^137^Cs source [Institute of Toxicology and Environmental Health, University of California, Davis; dose rate (R/min)=537.6 for a total of 9.7min]. At the indicated times after completion of irradiation, seedlings were snap frozen with liquid nitrogen. Histones were acid extracted as previously described (Friesner et al., 2005) and extracts run on 15% polyacrylamide gels and then transferred to PVDF membranes (Immobilon, Millipore Sigma). γ-H2AX was detected with rabbit anti-γ-H2AX (Sigma, product # H5912) and an anti-rabbit-HRP secondary antibody (Invitrogen, product #A16096).

## Results

### Transcriptional Profiling of *xct* Mutants Reveals Significant Effects on Circadian Phase of Gene Expression and Upregulation of Immune-Related Genes

XAP5 CIRCADIAN TIMEKEEPER was originally identified in a screen for *Arabidopsis* mutants with altered free-running circadian rhythms (Martin-Tryon et al., 2007). *xct-1* is a reduction-of-function allele predicted to produce a protein with an internal deletion of three amino acids, while *xct-2* is a presumptive null allele with a T-DNA inserted within an exon (Martin-Tryon and Harmer, 2008). Both alleles have a shorter free-running circadian period than wild-type and exhibit altered responsiveness to light. While most plant clock genes are specific to the green lineage, XCT encodes a protein highly conserved across eukaryotes but with no identifiable functional domains (Martin-Tryon and Harmer, 2008).

To better understand the molecular function of XCT, we carried out gene expression profiling on wild-type, *xct-1*, and *xct-2* seedlings. The circadian clock has pervasive effects on gene expression, regulating the expression of a large fraction of the *Arabidopsis* transcriptome (Harmer et al., 2000; Covington et al., 2008). Consequently, hundreds of genes are differentially expressed even within a single genotype when samples harvested at different times of day (and thus at different circadian phases) are compared to each other (Hsu and Harmer, 2012). Since *xct* mutants have a shorter free-running circadian period than wild-type (Martin-Tryon and Harmer, 2008), mutant and wild-type plants collected at the same time of day could potentially have differences in gene expression simply due to differences in circadian phase at the time of sample collection. To minimize this possibility, we grew plants in light/dark cycles and harvested them 3h after lights were turned on. The dark to light transition is a powerful clock-resetting cue in *Arabidopsis* (Covington et al., 2001), so we anticipated the modest differences in circadian phase (*ca.* 30min) between genotypes harvested at this time would have negligible effects on levels of gene expression. RNA-seq analysis was performed using three biological replicates, and differentially expressed genes were determined using the TAIR10 genome annotation and the Bioconductor package edgeR (Robinson et al., 2010) with the FDR controlled to below 5%.

Using these parameters, we found hundreds of genes misregulated in the two *xct* mutants when compared to wild-type (Supplementary Table 1). In both genotypes, many more of the differentially expressed genes are circadian-regulated than the ~30% expected by chance (Covington et al., 2008; 54% of genes differentially expressed in *xct-2* and 71% of genes differentially expressed in *xct-1* were previously defined as clock controlled (Hsu and Harmer, 2012); *p* values in both cases <2.2 e^−16^, Fisher’s exact test; Supplementary Figure S1A). This overrepresentation might be due either to preferential regulation of clock-controlled genes by XCT or to differences in circadian phase between the samples, with the short-period mutants being harvested at an earlier subjective time of day than the controls. In the latter case, we would predict that day-phased genes would have higher levels and night-phased genes would have lower levels of expression in the mutants relative to wild-type. This is exactly what we saw in both mutants: most upregulated genes that are also clock-regulated have peak circadian phases 6–12h after subjective dawn, while most downregulated genes that are also clock-regulated have peak circadian phases during the subjective night (Supplementary Figures S1B,C; Supplementary Table 1). In addition, the mean circadian rhythmic amplitudes of the genes differentially expressed in the mutants are significantly higher than mean rhythmic amplitude of all cycling genes (mean amplitudes for clock regulated gene differentially expressed in *xct-2*=0.394 and in *xct-1*=0.529; mean amplitude for all clock-regulated genes=0.255; *p*<2.2 e^−16^, KS test). These data suggest that a considerable fraction of gene misregulation in these mutants is due to differences in subjective circadian phase between the genotypes at the time of harvesting.

**Figure 1:**
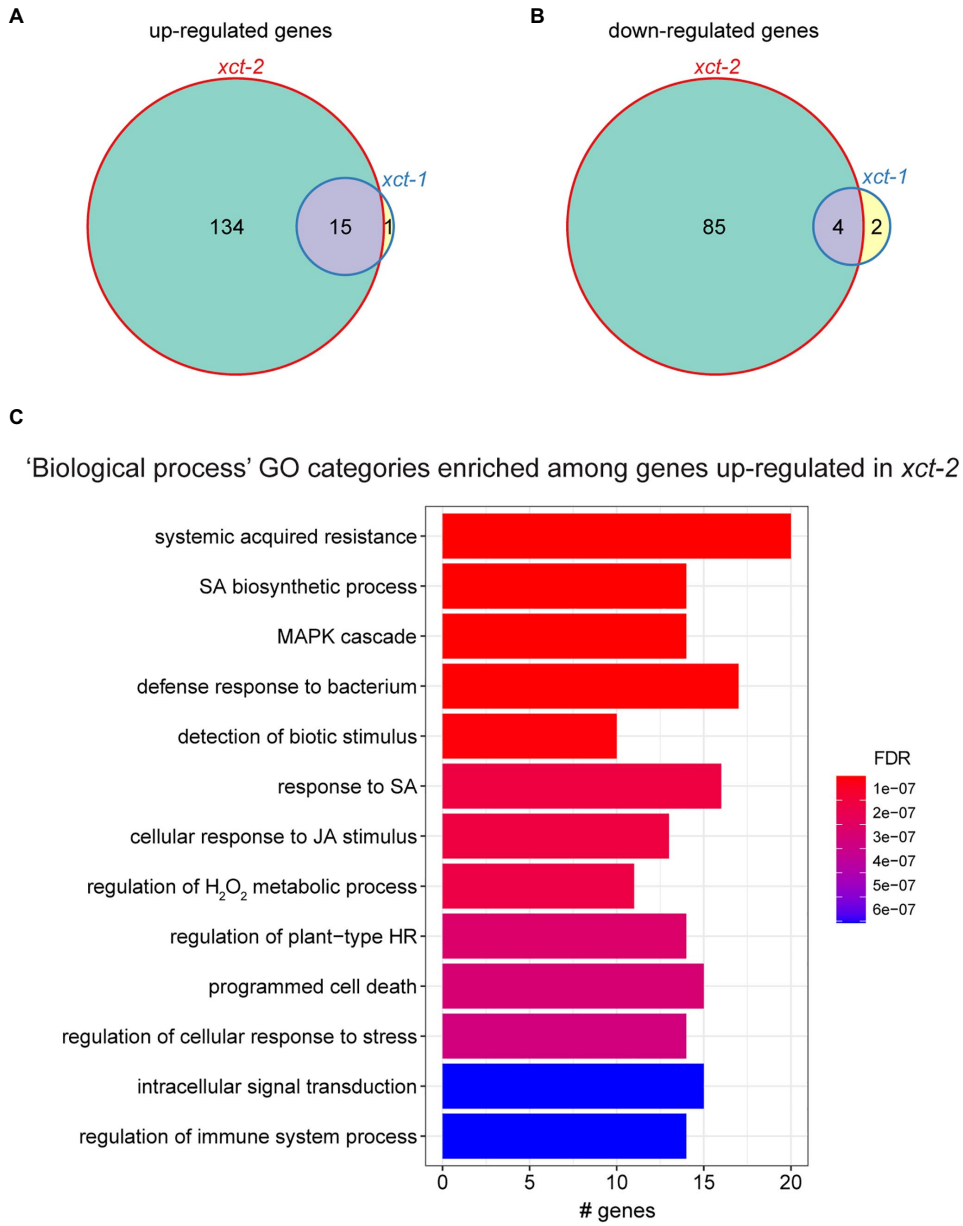
Immune-related genes are upregulated in *xct-2*. **(A,B)** Genes significantly differentially **(A)** upregulated or **(B)** downregulated in *xct-1* or *xct-2* plants as determined by RNA-seq [false discovery rate (FDR)<0.05 and a greater than 2-fold change relative to wild-type]. Genes previously defined as circadian regulated were omitted from this analysis. **(C)** The most significantly enriched Gene Ontology (GO) categories among genes upregulated in *xct-2* as determined using the clusterProfiler R package (Yu et al., 2012). (FDR<1 e-5; BH correction). No GO categories are significantly enriched among genes upregulated in *xct-1* or downregulated in either mutant.

Since it was not possible to determine whether changes in expression of clock-regulated genes in the *xct* mutants are due to the effect of XCT on clock pace or other possible roles for XCT, we excluded all ~6,600 genes, we previously defined as clock-regulated (Hsu and Harmer, 2012) from our subsequent analyses. After this filtering step, we found many more genes misexpressed in *xct-2* than in *xct-1*, with more transcripts upregulated than downregulated in both mutants (Figures 1A,B). Thus our data show the influence of XCT on gene expression extends beyond circadian regulation.

XAP5 CIRCADIAN TIMEKEEPER has previously been reported to promote the production of small RNAs *via* the regulation of transcription of the DICER-LIKE genes *DCL1*, *DCL3*, and *DCL4*, leading to upregulation of transcripts negatively regulated by small RNAs in *xct* mutants (Fang et al., 2015). We therefore investigated whether targets of small RNAs are enriched among genes upregulated in our *xct* transcriptomes by comparing genes differentially expressed in *xct* mutants and those previously reported to be targets of siRNAs or miRNAs (Addo-Quaye et al., 2008; German et al., 2008). We found no statistically significant overlap between genes differentially expressed in *xct* mutants and those regulated by small RNAs. Moreover, none of the small RNA-related genes previously reported to be significantly up- or downregulated in *xct* mutants as determined by qRT-PCR (Fang et al., 2015) are significantly differently expressed in *xct* mutants compared to wild-type in our RNA-seq analysis (Supplementary Figures S2A,B). Our inability to reproduce the findings reported by Fang et al. (2015) may either be due to different growth conditions between the labs or greater sensitivity of the qRT-PCR assay relative to our transcriptome analysis. But at any rate, these data suggest that the global gene expression phenotypes we observe in *xct* mutants are largely independent of altered production of small RNAs.

To better understand the biological processes affected by XCT, we examined the types of genes up- or downregulated in *xct* mutants. We found that genes upregulated in *xct-2* are significantly enriched for many Gene Ontology (GO) classifications related to plant defense responses (Figure 1C). In contrast, we found no significant enrichment for any GO categories in genes downregulated in *xct-2*, nor for genes up- or downregulated in *xct-1*. Inspection of genes significantly upregulated in *xct-2* and *xct-1* revealed a number of pathogenesis-related genes including *EARLY ARABIDOPSIS ALUMINUM INDUCED 1* (*EARLI1*), *EF-TU RECEPTOR* (*EFR*), *FLAGELLIN-SENSITIVE 2* (*FLS2*), and *MYB DOMAIN PROTEIN 95* (*MYB95*; Supplementary Figure S2C). Additional immune-related genes such as *PHYTOALEXIN DEFICIENT3* (*PAD3*) and *WRKY DNA-BINDING PROTEIN 70* (*WRKY70*) are upregulated in *xct-2* but not *xct-1* (Supplementary Figure S2C). All of these genes have previously been reported to be upregulated in response to pathogens or other defense-related treatments such as SA (Zhou et al., 1999; Eulgem et al., 2004; Li et al., 2004; Zipfel et al., 2006; Ma and Bohnert, 2007).

**Figure 2:**
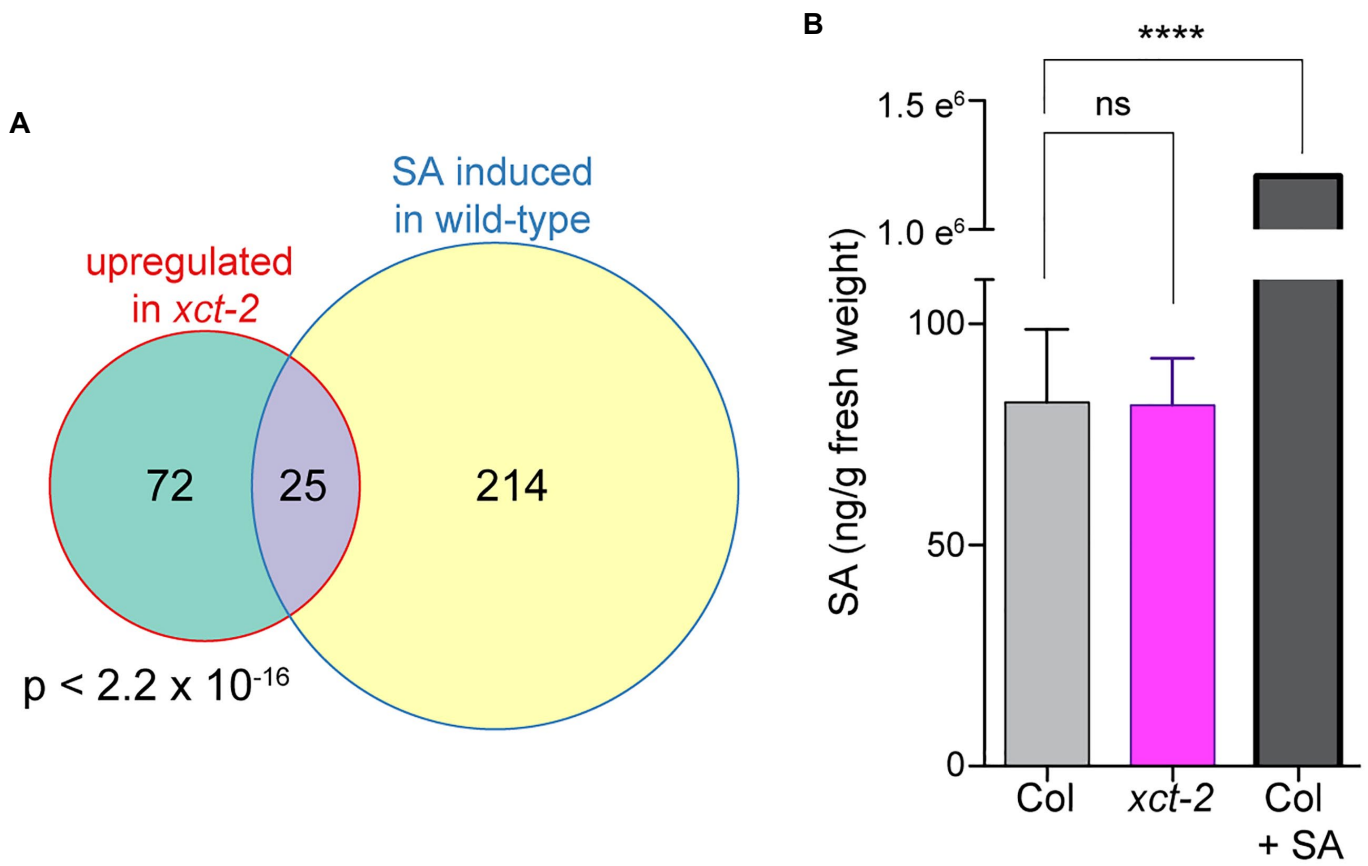
Increased basal expression of salicylic acid (SA)-induced genes in *xct-2* is not due to elevated SA levels. **(A)** The overlap between genes induced in *xct-2* relative to wild-type and genes upregulated in response to SA treatment (Pajerowska-Mukhtar et al., 2012) is highly statistically significant (Fisher’s exact test). Only genes expressed in both experiments and not classified as regulated by the circadian clock were considered. **(B)** Salicylic acid levels were determined using liquid chromatography-tandem mass spectrometry. ^****^p<0.0001; one-way ANOVA with Šidák correction for multiple comparisons. Data are representative of three biological replicates and are shown as±SEM (*n*=6).

We next performed a comparison between genes upregulated in *xct* mutants and those previously reported to be regulated by SA (Pajerowska-Mukhtar et al., 2012). Confining our analysis to transcripts detectably expressed in both experiments, we found a highly statistically significant overlap between genes upregulated in *xct-2* and those induced in response to SA (Figure 2A). This suggested that basal SA levels might be constitutively increased in the *xct* mutant. However, we found instead that SA levels in *xct-2* seedlings are the same as those of wild-type controls (Figure 2B). These data suggest that immune signaling pathways in *xct* are activated independently of increased SA biosynthesis.

### Enhanced Immune Responses in *xct* Mutants Are Temperature- and *npr1*-Independent

**Figure 3:**
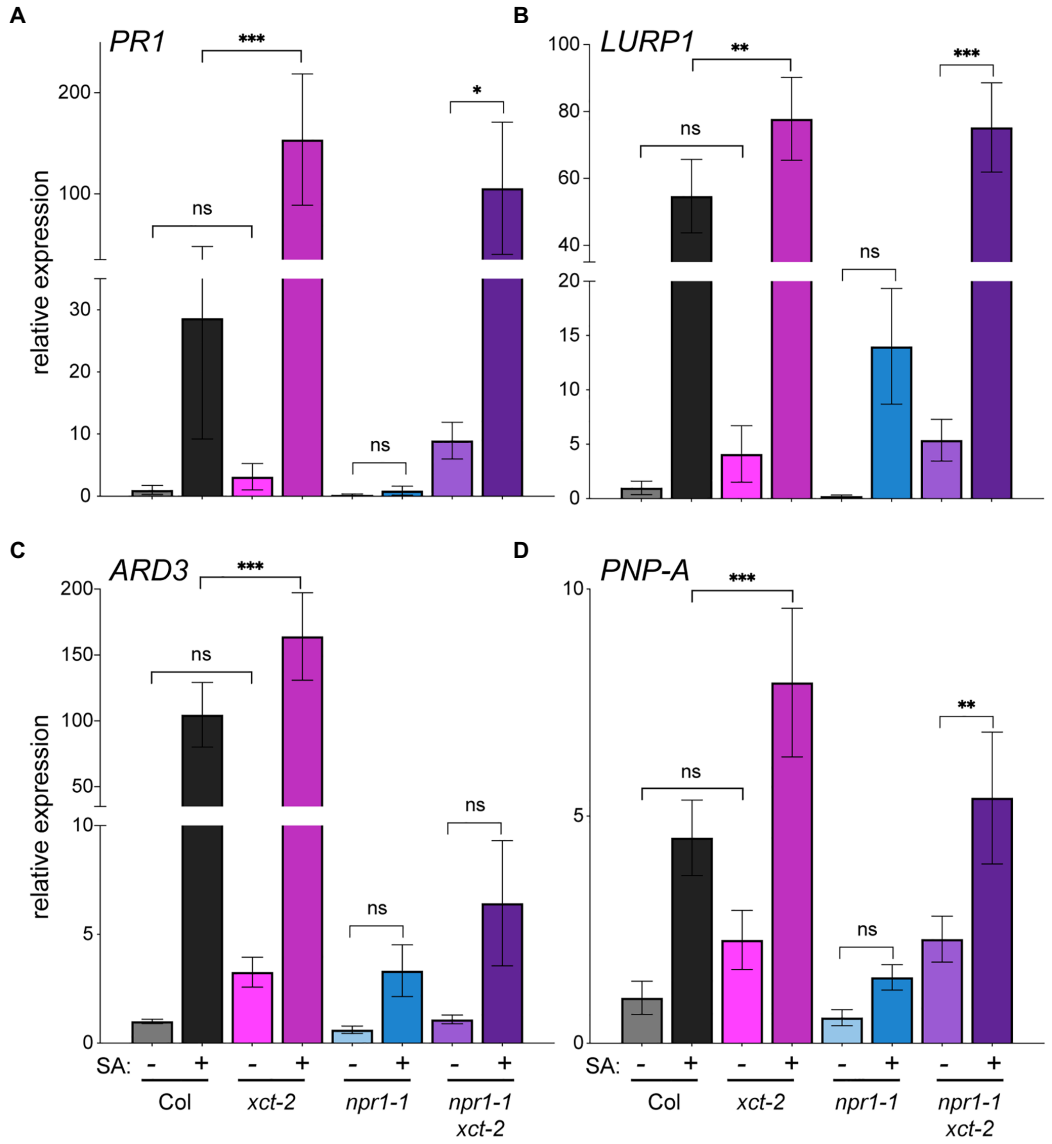
Loss of XAP5 CIRCADIAN TIMEKEEPER (XCT) function restores salicylic acid responsiveness to *npr1* mutants. **(A–D)** Expression of pathogenesis-related genes in Col, *xct-2*, *npr1-1*, and *npr1-1 xct-2* that were either mock-treated or sprayed with 0.3mM salicylic acid as determined by quantitative reverse-transcriptase PCR (qRT-PCR). Samples were collected 24h after treatment. Expression levels of the indicated genes were determined by qRT-PCR and normalized to *PP2C*. Means of three technical replicates are plotted, ±SD. ^*^p<0.05; ^**^p<0.005; ^***^p<0.001; and one way ANOVA with Šidák correction for multiple comparisons. Data are representative of three biological replicates.

To further investigate immune-related phenotypes in *xct* mutants, we next examined expression of pathogenesis-related genes before and after treatment with SA. We used qRT-PCR to quantify expression of several genes previously reported to be upregulated in response to pathogens: *PATHOGENESIS-RELATED1* (*PR1)*, *LATE UPREGULATED IN RESPONSE TO HYALOPERONOSPORA PARASITICA1* (*LURP1*), *ACIREDUCTONE DIOXYGENASE 3* (*ARD3*), and *PLANT NATRIURETIC PEPTIDE A* (*PNP-A*; Eulgem et al., 2004; Huang et al., 2005; Ascencio-Ibanez et al., 2008; Knoth and Eulgem, 2008; Ficarra et al., 2018). Although mean basal levels of these genes trend higher in *xct-2* than wild-type, the differences between the genotypes did not reach statistical significance (Figure 3; Supplementary Figure S3). However, comparison of expression levels between SA-treated wild-type and *xct-2* plants revealed significantly higher SA-induced expression levels in the mutant than in Col (Figure 3). This result was confirmed with further statistical analysis using linear mixed-effect models that show a significant effect of the *xct-2* genotype on responsiveness to SA for *PR1*, *LURP1*, and *PNP-A* (Supplementary Figure S3). Thus loss of XCT function potentiates plant transcriptional responses to SA.

We next wanted to determine where XCT acts in SA signaling relative to NPR1, an important SA receptor (Wu et al., 2012; Ding et al., 2018; Wang et al., 2020). As expected, we found induction of pathogenesis-related genes in response to SA to be strongly blunted in *npr1-1* mutants, with no statistically significant difference in expression levels differences before and after treatment (Figure 3). Intriguingly, induction of gene expression in response to SA is largely restored in *npr1-1 xct-2* mutants treated with SA (Figure 3; Supplementary Figure S3). These data suggest that XCT affects immune signaling independently of the key SA signaling component NPR1.

**Figure 4:**
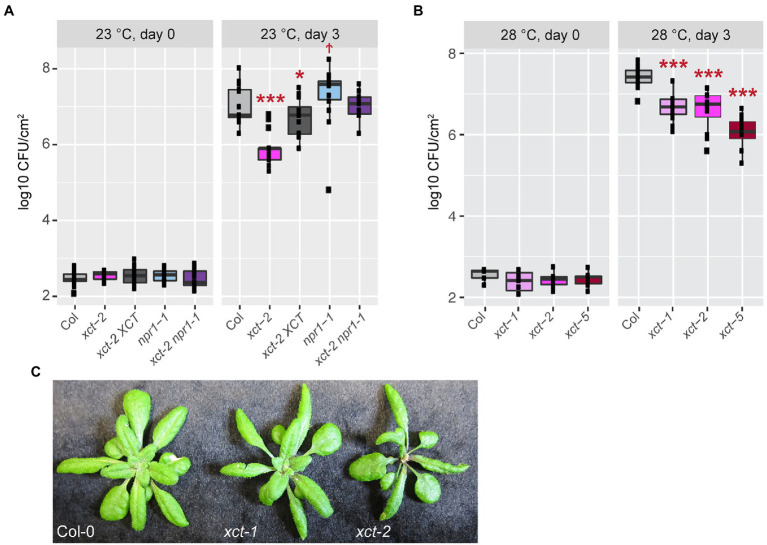
Enhanced immune responses in *xct* mutants are independent of temperature. The indicated genotypes were grown at either 23 **(A)** or 28°C **(B)** and inoculated with *Pseudomonas syringae* pv. *tomato* DC3000. Bacterial growth was measured at the indicated time points; titers are represented as log colony forming units per cm^2^ (log CFU/cm^2^) of leaf tissue. Data are from three **(A)** or two **(B)** independent experiments (*n*=4–8 plants per replicate). The lines within the boxes are the medians and the upper and lower hinges represent the first and third quartiles. Statistical significance of mutant values relative to wild-type was determined using linear mixed regression models with genotype as fixed effect, trial as random effect (^†^*p*=0.06; ^*^p<0.05; and ^***^p<1e^−4^). **(C)** Plants of the indicated genotypes were grown at 22°C.

Increased expression of pathogenesis-related genes, for example in response to a previous infection or treatment with a defense elicitor, can increase disease resistance. We therefore next investigated the susceptibility of *xct* mutants to the bacterial pathogen *Pseudomonas syringae* pv. *tomato* DC3000. Leaves were inoculated with this virulent pathogen and bacterial growth was assessed after 3days. We found that *xct-2* mutants maintained at 23°C are highly resistant, with bacterial titers 10-fold lower than seen in wild-type controls (Figure 4A). The *xct-2* resistance phenotype is largely but not completely suppressed by introduction of XCT-YFP-HA expressed under the control of the endogenous *XCT* promoter (Figure 4A; Supplementary Figure S4A). Similar to the partial rescue seen for the pathogen resistance phenotype, this line also exhibits partial rescue of the *xct-2* short-period circadian clock phenotype (Supplementary Figure S5).

Given that *xct-2* restores SA responsiveness to *npr1* mutants (Figure 3; Supplementary Figure S3), we examined disease resistance in *xct-2 npr1* mutants as well. As previously reported (Cao et al., 1994; Glazebrook et al., 1996), we found *npr1* mutants are more susceptible to bacterial infection than controls, albeit with a relatively low statistical significance in our experiments (Figure 4A). Intriguingly, we found that *npr1 xct* double mutants have an additive phenotype, showing similar bacterial titers 3days after inoculation as wild-type controls (Figure 4A; Supplementary Figure S4A). Thus our genetic studies reveal that XCT is a negative regulator of plant immunity that acts in an NPR1-independent manner.

One possible cause for this increased disease resistance could be constitutive activation of ETI signaling in *xct* mutants. This type of immunity is almost always associated with increased levels of SA and the formation of spontaneous lesions (Durner et al., 1997; Balint-Kurti, 2019). However, we did not observe visible lesions in *xct-1* or *xct-2* plants in any of our growth conditions, and trypan blue staining did not reveal spontaneous necrosis (Supplementary Figure S4C and data not shown). Constitutive ETI is also temperature sensitive, being most obvious at low temperature such as 16°C and suppressed at 28°C (Yang and Hua, 2004; Wang et al., 2009; Kim et al., 2010; Zhu et al., 2010; Alcazar and Parker, 2011). We therefore investigated the susceptibility of wild-type, *xct-1*, *xct-2*, and the recently reported allele *xct-5* (Xu et al., 2017) to *Pseudomonas syringae* pv. *tomato* DC3000 in plants maintained at 28°C. We found that all three *xct* alleles have a similar degree of resistance to this pathogen at 28°C as observed at 23°C (Figure 4B; Supplementary Figure S4A). As expected, bacterial growth was generally promoted at the higher temperature (Supplementary Figure S4B).

Another trait seen in mutants with constitutive activation of ETI is reduced growth relative to wild-type plants (Balint-Kurti, 2019). We therefore assessed rosette size and plant morphology in *xct* mutants. The rosette diameter of *xct-2* plants is somewhat smaller than that of control plants, but *xct-1* and Col-0 plants have very similar morphology and overall size (Figure 4C). *xct-1* and *xct-2* have similar enhanced resistance phenotypes (Figure 4B), indicating that the growth and immune phenotypes of *xct-2* are separable. Overall, the absence of spontaneous lesions, lack of elevated SA levels, temperature independence of the pathogen resistance phenotype, and normal size of the pathogen-resistant *xct-1* plants suggest that pathogen resistance in *xct* mutants is not caused by a classic constitutive activation of immunity phenotype.

XAP5 CIRCADIAN TIMEKEEPER has previously been reported to affect immune responses *via* positive regulation of the ectopic expression of the resistance gene *RPW8*.1 (Xu et al., 2017), which is normally not expressed in the Col-0 accession (Xiao et al., 2001). However, Xu et al. (2017) found that *xct-5* mutants in Col-0 plants not expressing *RPW8.1* have the same susceptibility to a virulent bacterial pathogen as wild-type controls. In contrast, we found in repeated experiments that *xct-5* and other *xct* alleles are more resistant than wild-type (Figure 4B). The reason for this discrepancy is not clear. Even though we found disease resistance in *xct* mutants to be robust at different temperatures, it is possible that other environmental variables could affect this phenotype.

### XCT Plays a Role in DNA Damage Response Pathways

**Figure 5:**
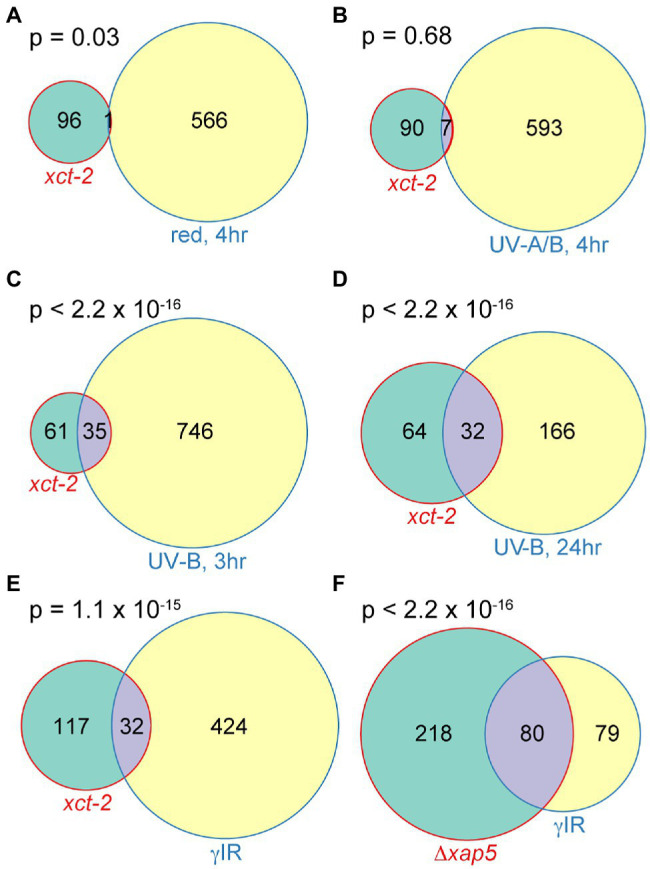
Significant overlaps between genes upregulated in *xct-2* and those induced in response to DNA damaging treatments in *Arabidopsis* and fission yeast. **(A–E)** Overlaps between genes induced 2-fold or more in *xct-2* relative to wild-type and genes upregulated 2-fold or more in plants exposed to **(A)** 10 μmolm^−2^ s^−1^ red light for 4h, **(B)** 7W m^−2^ UV-A/B light for 4h, **(C,D)** treated with 1.2W m^−2^ UV-B light and harvested either 3 or 24h later, or **(E)** treated with 100Gy of γ radiation. Light-induced genes in **(A,B)** were reported by Peschke and Kretsch (2011), UV-B induced genes **(C,D)** were reported by Kilian et al. (2007), and γ radiation-induced genes **(E)** correspond to those assigned to paths W1–W4 by Bourbousse et al. (2018). Only genes not classified as regulated by the circadian clock (Hsu and Harmer, 2012) and expressed both in our RNA-seq experiment and the previously published transcriptome experiments were considered. **(F)** Significant overlap between genes upregulated in *S. pombe Δxap5* mutants relative to wild-type (Anver et al., 2014) and those induced in response to γ ionizing radiation (Watson et al., 2004). Statistical significance of overlapping genes was determined using Fisher’s exact test.

Since our data suggest XCT plays an unconventional role in immune signaling, we examined previously-published transcriptome data for further clues to its molecular function. We previously found *xct* mutants are hypersensitive to light input to the circadian clock (Martin-Tryon and Harmer, 2008). Since activation of light signaling pathways potentiates plant defenses (Ballare, 2014), we compared genes upregulated in *xct* mutants and those induced in response to light. Limiting our analysis to transcripts expressed in both our experiment and the previously-published datasets, we found no significant enrichment of genes differentially expressed in *xct-2* and those induced in response to either monochromatic (blue, red, far-red, UV-A, or UV-A/B) or white light (Peschke and Kretsch, 2011; Figures 5A,B; Supplementary Table 2). These transcriptome data suggest that photomorphogenetic signaling pathways are not constitutively activated in *xct* mutants.

In contrast, we did find highly statistically significant overlaps between genes upregulated in *xct-2* and those induced in response to damaging levels of UV-B light (Kilian et al., 2007; Figures 5C,D; Supplementary Table 2). The degree of overlap between genes upregulated in *xct-2* and those induced by high levels of UV-B light is greater at later vs. earlier time points post irradiation. This, combined with the lack of enrichment between genes induced in response to photomorphogenetic light treatments, suggests the overlap may be due to genes induced in response to cellular damage rather than due to activation of fast-acting light response pathways.

Damaged DNA has previously been reported to potentiate immune responses in animals and plants (Yan et al., 2013; Gallucci and Maffei, 2017). We therefore investigated the degree of overlap between genes upregulated in *xct-2* and those displaying a rapid and sustained increase in expression after γ irradiation (Bourbousse et al., 2018). We found a highly statistically significant overlap in genes differentially expressed in these two experiments (Figure 5E). Since we previously found that *Arabidopsis* XCT and *S. pombe xap5* are functional orthologs (Anver et al., 2014), we next examined whether there is a significant overlap between genes misregulated in *S. pombe Δxap5* mutants and those induced in wild-type yeast subjected to γ irradiation (Watson et al., 2004). We found a highly statistically significant overlap between these two gene sets as well (Figure 5F). Together, these transcriptomic analyses suggest that *XCT* deficiency in plants or *xap5* deficiency in fission yeast might cause alterations in cellular responses to DNA damaging agents, an intriguing possibility given previous reports of the involvement of DNA damage response signaling factors in plant defense responses (Durrant et al., 2007; Wang et al., 2010; Song et al., 2011; Yan et al., 2013; Bourbousse et al., 2018; Ogita et al., 2018).

**Figure 6:**
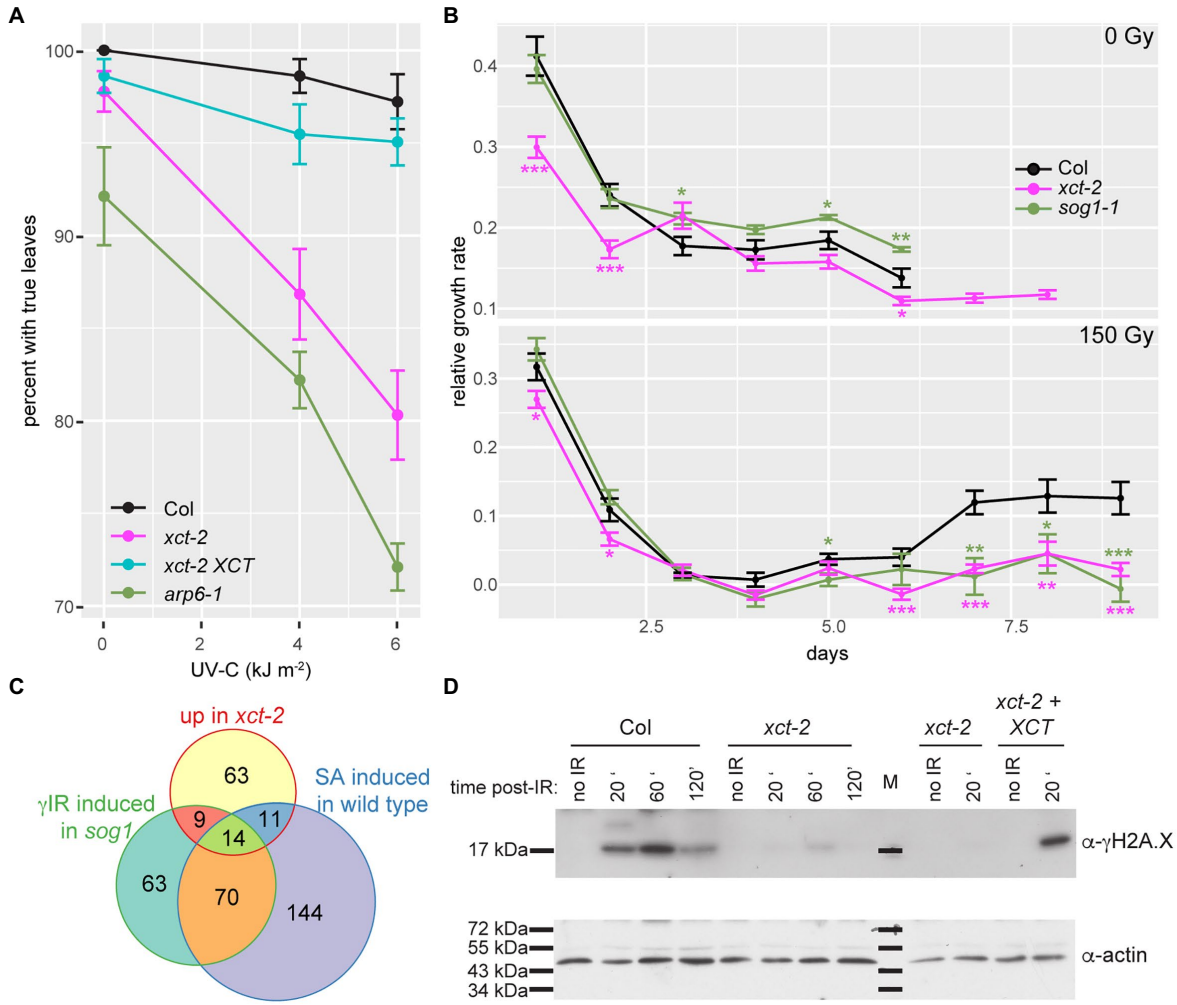
*xct-2* mutants are deficient in response to DNA damaging agents. **(A,B)** Growth of *Arabidopsis* seedlings was assessed after UV-C or γ irradiation. **(A)** Formation of true leaves was determined for plants of the specified genotypes 7days after exposure to the indicated fluences of UV-C irradiation. Using a linear mixed effect model with genotype and irradiance as fixed effects and trial as random effect, the genotype by irradiance interaction was found to be significant for *xct-2* (*p*=1.2 e-5) and for *arp6-1* (*p*=1.0 e-6) but not for *xct-2 XCT* (*p*=0.74). **(B)** Relative growth rate (Hoffmann and Poorter, 2002) of primary roots was determined for control seedlings and seedlings treated with 150Gy of γ irradiation. Asterisks indicate statistically significant differences from wild-type (Col); ^*^p<0.05; ^**^p<0.01; and ^***^p<0.001 (Student’s *t*-test), *n*=13–29. **(C)** Statistically significant overlap between genes induced 2-fold or more in *xct-2* relative to wild-type, genes upregulated 2-fold or more in wild-type plants treated with SA (Pajerowska-Mukhtar et al., 2012), and *sog1* mutants treated with 100Gy of γ radiation (Bourbousse et al., 2018). Only genes expressed in all three experiments and not classified as regulated by the circadian clock were considered. All three two-way comparisons are highly significant (*p*<2.2 e^−16^, Fisher’s exact test). **(D)** Levels of histone H2A.X phosphorylation in seedlings after treatment with 50Gy of γ radiation were assessed by blotting nuclear extracts with a γH2A.X-specific antibody. Plants were harvested at the indicated times after irradiation. “M” indicates molecular weight marker. The data are representative of three biological replicates.

We therefore next investigated whether responses to DNA damaging treatments are in fact altered in *xct*. In *Arabidopsis*, genotoxic treatments cause DNA-damage-dependent G_2_ cell cycle arrest in stem cells, inhibiting leaf development and root elongation (Preuss and Britt, 2003; Johnson et al., 2018). We first examined whether loss of *XCT* affects plant susceptibility to UV-C irradiation in the shoots. We found that *xct-2* mutants are significantly more susceptible to UV irradiation than wild-type, with considerable inhibition of true leaf production in *xct-2* at UV-C doses that have little effect on controls (Figure 6A; Supplementary Figure S6A). Similar effects of UV-C on true leaf formation are seen in *arp6* mutants, consistent with previous reports (Rosa et al., 2013), while an *XCT* transgene rescues the *xct-2* phenotype.

Next, we assessed primary root elongation after treatment of seedlings with levels of ionizing radiation that cause cell cycle arrest and programmed cell death in the root apical meristem, halting root elongation until the meristem undergoes regeneration (Johnson et al., 2018). These processes are largely dependent upon *SOG1*, a plant-specific gene encoding a transcription factor that is a central mediator of DNA damage response pathways (Preuss and Britt, 2003; Yoshiyama et al., 2009; Johnson et al., 2018). As previously reported, root elongation in wild-type plants ceases by 3days after irradiation with 150Gy but recovers a few days later (Figure 6B). In contrast, while root growth in *sog1-1* and *xct-2* plants shows a similar acute response to the DNA damaging treatment, elongation of the primary root does not resume in either of these mutants by 9days after irradiation (Figure 6B). Thus *xct* mutants and *sog1* mutants show very similar susceptibility to DNA-damaging γ irradiation.

Although the DNA damage response is strongly perturbed in *sog1* mutants, it is not entirely absent: a subset of genes induced in response to γ irradiation in wild-type are also induced in *sog1* mutants, albeit with slower kinetics (Bourbousse et al., 2018). It has been posited that this slow and *SOG1*-independent upregulation is triggered by unrepaired DNA lesions. Since it has been suggested that damaged DNA potentiates immune responses (Yan et al., 2013), we next examined the overlap between these *sog1*-independent, γ irradiation-induced genes, and those upregulated either in response to the immune elicitor SA (Pajerowska-Mukhtar et al., 2012) or in *xct-2* mutants. We found that the majority of genes upregulated after γ irradiation in *sog1* mutants are also induced in response to SA. In addition, more than a third of the genes upregulated in *xct-2* are induced in response to SA and/or upregulated in γ irradiated *sog1* mutants (Figure 6C). These similarities between the three transcriptome profiles further suggest functional links between XCT, immune signaling, and DNA damage response pathways.

SUPPRESSOR OF GAMMA RESPONSE 1 activity is dependent upon phosphorylation by ATM kinase (Yoshiyama et al., 2013, 2017), which along with the related kinase ATR is rapidly activated after DNA damaging treatments. Another important substrate of these kinases is the histone variant H2A.X, which when phosphorylated on serine 139 acts as a scaffold to assemble DNA damage response factors at sites of DNA damage (Ciccia and Elledge, 2010). In *Arabidopsis*, both ATM and ATR mediate rapid phosphorylation of H2A.X after DNA damaging treatments (Friesner et al., 2005). Detection of this phosphorylated form of H2A.X, termed γ-H2A.X, is frequently used as a proxy to detect activation of DNA damage response signaling pathways in response to double strand breaks.

To assess early DNA damage response signaling processes in *xct*, we examined overall levels of γ-H2A.X in extracts made from plants before and after γ irradiation. Consistent with previous reports (Friesner et al., 2005), we did not detect γ-H2A.X in extracts made from unirradiated plants. However, treatment of wild-type plants with 50Gy of ionizing radiation resulted in the appearance of an anti-γ-H2A.X reactive band. This band was strongest in samples harvested 60min after the end of irradiation but was evident at earlier and later time points as well (Figure 6D). In *xct-2* mutants, in contrast, almost no γ-H2A.X signal could be detected after irradiation (Figure 6D; Supplementary Figure S6B). This loss of reactivity was rescued by an *XCT* transgene, demonstrating that mutation at the *XCT* locus is responsible for this phenotype.

This profound loss of H2A.X phosphorylation after irradiation is stronger than the decrease reported for single *atm* or *atr* mutants but similar to that reported for *atm atr* double mutants (Friesner et al., 2005). These data demonstrate that *xct* mutants are deficient in either the detection of or early response to DNA damaging ionizing radiation. In addition, these results suggest that the greater susceptibility of *xct* to genotoxic treatments is due to a specific defect in DNA damage response pathways rather than a general effect on plant health.

## Discussion

XAP5 CIRCADIAN TIMEKEEPER was first identified in a screen for plants with alterations in circadian clock function (Martin-Tryon and Harmer, 2008). Here, we used a transcriptomic approach to gain further insight into XCT function in *Arabidopsis*, collecting tissue samples from wild-type and mutant plants at a single time point. Since perturbation of many signaling pathways can affect the period and amplitude of circadian clock-regulated gene expression (Sanchez and Kay, 2016), additional discussion of our experience with transcriptional profiling of *xct* mutants may be useful to those working in a variety of fields. In *Arabidopsis*, the circadian clock is set by imbibition and considerable synchrony is maintained across individuals and even populations after weeks in constant environmental conditions (Salome et al., 2008; Wenden et al., 2012; Greenwood et al., 2019). Therefore, harvesting single tissue samples from plants grown in constant environmental conditions, even for extended periods of time, does not ensure arrhythmicity and possible circadian differences between genotypes must still be considered. Instead of this approach, we maintained all genotypes in entraining conditions and harvested them all soon after lights on. We reasoned that the small differences in internal circadian phase between the genotypes at the time of harvest would have only modest effects on gene expression levels. We were surprised to find, however, that the majority of differentially expressed genes in both *xct* mutants showed circadian phase and amplitude characteristics (Supplementary Figure S1) similar to genes, we previously found to be differentially expressed between samples of wild-type plants harvested at different times of day (Hsu and Harmer, 2012). We conclude that a more effective method to minimize effects of different rhythmic amplitudes or free-running periods on gene expression analysis would be to harvest samples from the genotypes of interest at multiple times during the subjective day and night and then pool different time points before analysis, and we recommend this approach to others.

Exclusion of clock-regulated transcripts from our differential expression analysis allowed us to identify alterations in responses to DNA damaging treatments (Figure 6; Supplementary Figure S6) and SA (Figure 3; Supplementary Figure S3) and increased pathogen resistance in *xct* mutants (Figure 4; Supplementary Figure S4). Increased pathogen resistance is frequently found in mutants with constitutive activation of ETI, either due directly to loss-of-function of negative regulators of this process or to loss of a target of pathogen secreted virulence factor “guarded” by immune signaling components (Chakraborty et al., 2018). Indeed, the elevated basal levels of some defense genes in *xct-2* suggest this might be the case (Figure 1; Supplementary Figure S2). However, other lines of evidence suggest the resistance phenotypes in *xct* mutants are not due to constitutive ETI. Mutants with constitutive ETI typically display spontaneous lesions, elevated levels of SA, and temperature-dependent pathogen resistance (Durrant et al., 2007; Van Wersch et al., 2016; Chakraborty et al., 2018), phenotypes that we do not observe in *xct* (Figures 2, 4; Supplementary Figure S4). Moreover, while constitutive ETI is commonly associated with strong inhibition of plant growth (Balint-Kurti, 2019), we find that *xct-1* mutants are very similar in size to wild-type controls, while *xct-2* mutants are only moderately smaller (Figure 4; Supplementary Figure S4). Finally, mutants with constitutive ETI display elevated basal levels of γ-H2A.X (Rodriguez et al., 2018), not the almost complete loss of γ-H2A.X after DNA damage that we observe in *xct* mutants (Figure 6; Supplementary Figure S6). Together, these data strongly suggest that the enhanced immunity observed in *xct* alleles is not due to classic constitutive ETI.

The increased susceptibility to DNA damaging treatments and highly reduced induction of γ-H2A.X after irradiation of *xct* mutants (Figure 6; Supplementary Figure S6) indicate that the DNA damage response pathway is compromised in these plants. DNA damage response signaling has long been linked to immunity in both plants and animals (Camborde et al., 2019; Nastasi et al., 2020). However, the relationship between DNA damage and plant immunity is complex and not fully resolved. Some report that treatment with the defense elicitor SA can cause damaged DNA (Yan et al., 2013; Hadwiger and Tanaka, 2017), while others have not found this to be the case (Song and Bent, 2014). Activation of ETI in the absence of pathogens causes the accumulation of damaged DNA, suggesting DNA damage during infection may be entirely a consequence of programmed host cell death (Rodriguez et al., 2018). However, the appearance of the DNA damage marker γ-H2A.X as soon as 2h after pathogen infection (much earlier than the first observable signs of plant cell death; Zhang et al., 2004; Song et al., 2015) suggest DNA damage may not just be an indirect product of the plant hypersensitive response. Indeed, damaged DNA (both foreign and host) has been reported to potentiate immune responses (Wen et al., 2009; Yakushiji et al., 2009; Gallucci and Maffei, 2017; Toum et al., 2020). Finally, the ability of DNA damaging treatments to promote innate immunity (Kunz et al., 2008; Yan et al., 2013) also suggests DNA damage and its consequent response pathways may activate immune signaling in plants.

DNA damage causes rapid activation of the ATM and ATR kinases; in plants, both kinases then phosphorylate H2A.X and other mediators of the DNA damage response pathway (Friesner et al., 2005; Ciccia and Elledge, 2010). The strong reduction in γ-H2A.X signal in irradiated *xct* mutants relative to wild-type (Figure 6; Supplementary Figure S6) suggests that the function of the ATM and ATR kinases may be perturbed in the absence of XCT. Given the requirement of ATM phosphorylation of SOG1 for DNA binding by this master regulator of DNA damage responses (Yoshiyama et al., 2013; Yoshiyama, 2016), this suggests that SOG1 function might also be perturbed in *xct* mutants. Intriguingly, gene expression in both plants overexpressing *SOG1* and in *sog1* null mutants is hyper-responsive to SA (Ogita et al., 2018; Yoshiyama et al., 2020), indicating that both increased and decreased SOG1 function increase plant responsiveness to this immune elicitor. Given the cross-regulation of defense-related and DNA damage response genes by SOG1, the DNA damage response phenotypes seen in *xct* mutants, and the overlaps between genes misexpressed in *xct-2* and those induced by SA and γ irradiation, it is tempting to speculate that the enhanced pathogen resistance in *xct* mutants may be secondary to a role for XCT in DNA damage response pathways. In support of this possibility, a recent study has found that XCT is rapidly phosphorylated in response to γ irradiation (Waterworth et al., 2019).

Although we originally isolated *XCT* based on the circadian phenotype of plants mutant for this gene, its high degree of conservation across eukaryotes suggest its primary function lies outside the circadian system (Martin-Tryon and Harmer, 2008; Hsu and Harmer, 2014). We previously reported that the *S. pombe* XCT homolog Xap5 has genetic interaction profiles similar to components of chromatin remodeling complexes and that Xap5 helps suppress expression of aberrant transcripts (Anver et al., 2014). This, along with the obvious effects of loss of *XCT* on DNA damage responses, leads us to suggest XCT proteins may play an important role in regulation of chromatin dynamics. This possibility awaits further, likely biochemical, studies.

### Accession Numbers

*Arabidopsis thaliana* genes referenced in this paper have the following accession numbers:

AGO1, AT1G48410.
ARD3, AT2G26400.
ARF3, AT2G33860.
DCL1, AT1G01040.
DCL3, AT3G43920.
DCL4, AT5G20320.
EARLI1, AT4G12480.
EFR, AT5G20480.
FLS2, AT5G46330.
LURP1, AT2G14560.
MYB65, AT3G11440.
MYB95, AT1G74430.
NPR1, AT1G64280.
PAD3, AT3G26830.
PNP-A, AT2G18660.
PR1, AT2G14610.
SNI1, AT4G18470.
SOG1, AT1G25580.
SPL3, AT2G33810.
TCP4, AT3G15030.
XCT, AT2G21150.
WRKY70, AT3G56400.

Short-read sequences are deposited in Gene Expression Omnibus (GEO) with accession number GSE67813.

## Data Availability Statement

The original contributions presented in the study are publicly available. This data can be found at: NCBI repository, accession number: PRJNA281004 (https://www.ncbi.nlm.nih.gov/geo/query/acc.cgi?acc=GSE67813).

## Author Contributions

RK, CE, TT, AB, HZ, CLC, GC, and SLH contributed to data analysis. RK performed the qRT-PCR and DNA damage susceptibility experiments. CE carried out the transcriptome analysis. TT conducted the pathogen resistance assays. AB quantified the salicylic acid levels. HZ carried out UV irradiation experiments. CLC, GC, and SLH supervised the experiments. SLH conceived the project, conducted the histone experiments, wrote the article with contributions from all authors, and agrees to serve as the author responsible for contact and ensures communication. All authors contributed to the article and approved the submitted version.

## Funding

This work was supported by awards from the National Institutes of Health (R01 GM069418 to SLH and R35GM136402 to GC), from the National Science Foundation (IOS 1723926 to CLC), and the United States Department of Agriculture-National Institute of Food and Agriculture (CA-D-PLB-2259-H to SLH).

## Conflict of Interest

The authors declare that the research was conducted in the absence of any commercial or financial relationships that could be construed as a potential conflict of interest.

## Publisher’s Note

All claims expressed in this article are solely those of the authors and do not necessarily represent those of their affiliated organizations, or those of the publisher, the editors and the reviewers. Any product that may be evaluated in this article, or claim that may be made by its manufacturer, is not guaranteed or endorsed by the publisher.

## Supplementary Material

Supplementary Figure 1 Skewed circadian phases in genes misregulated in *xct* mutants.

Supplementary Figure 2 Expression of small RNA- and immune-related genes in *xct* mutants.

Supplementary Figure 3 Loss of XCT function increases salicylic acid responsiveness.

Supplementary Figure 4 Enhanced immune responses in *xct* mutants are independent of temperature.

Supplementary Figure 5 Circadian period phenotypes of *xct* alleles.

Supplementary Figure 6 *xct-2* mutants are deficient in response to DNA damaging agents.

Supplementary Table 1 Differential gene expression for *xct-1* and *xct-2* compared to wild type.

Supplementary Table 2 Genes up-regulated in *xct-2* are enriched for regulation by γ irradiation and UV-B but not for regulation by non-damaging light treatments.

Supplementary Table 3 Primers used in this study.
