# Supplementary material for: XAP5 CIRCADIAN TIMEKEEPER Affects Both DNA Damage Responses and Immune Signaling in Arabidopsis
Source: Front Plant Sci. 2021 Oct 1;12:707923. doi: 10.3389/fpls.2021.707923 (PMC8517334; doi:10.3389/fpls.2021.707923)
Supplement: Supplementary Figure 1 — Skewed circadian phases in genes misregulated in xct mutants. [file Data_Sheet_1.PDF]

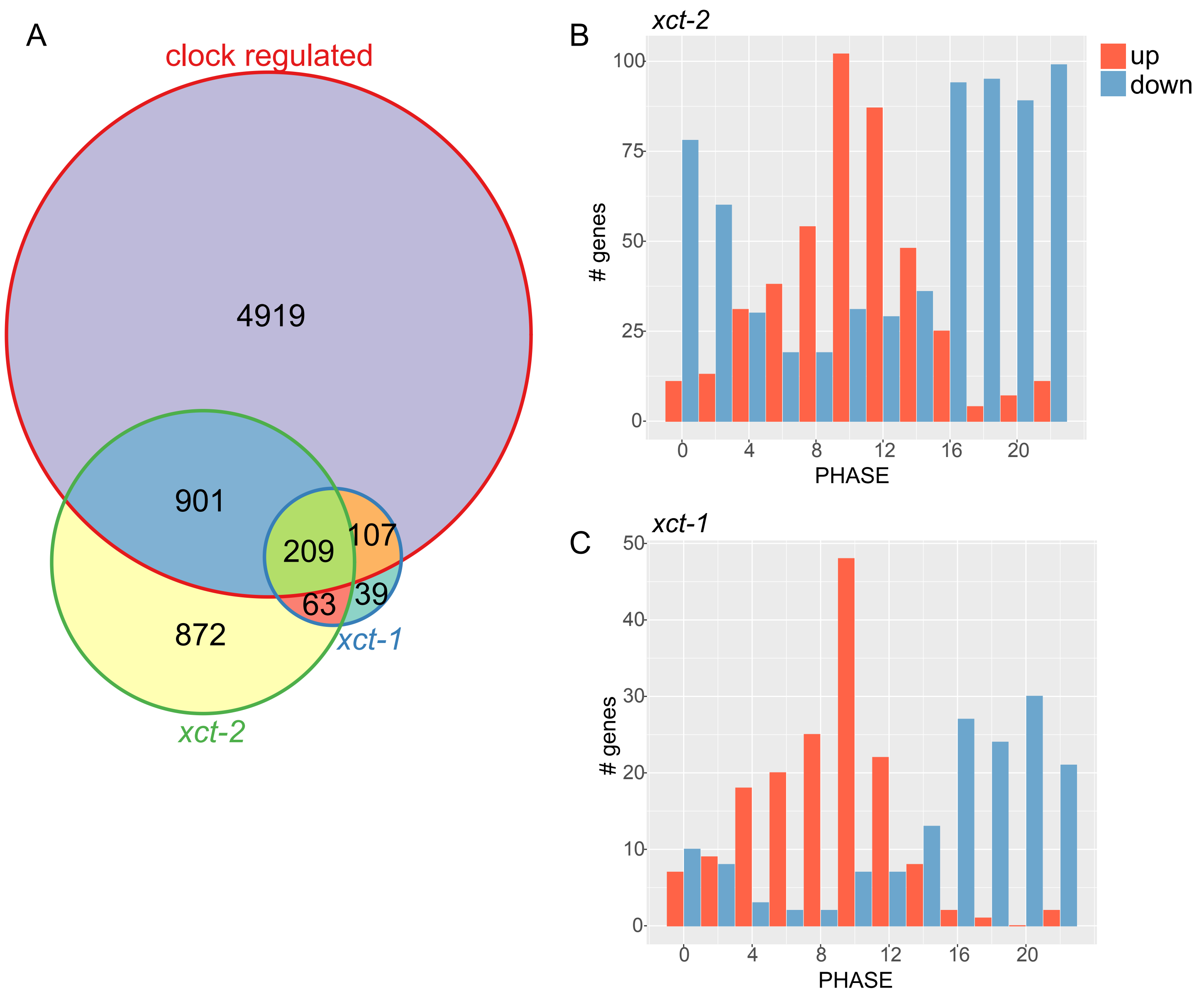

Supplementary Figure 1. Skewed circadian phases in genes misregulated in *xct* mutants.
(A) Extensive overlap between genes differentially expressed in the *xct-1* or *xct-2* mutants and those previously defined as clock-regulated by the JTK_Cycle algorithm (Hsu and Harmer, 2012). Genes not detectably expressed in both the current and previous RNA-seq experiments were excluded from this analysis. (B – C) The peak circadian phases of expression of genes significantly up- or down-regulated in the (B) *xct-2* or (C) *xct-1* mutants. Time 0 represents subjective dawn and 12 represents subjective dusk. The opposite phase distributions of up- and down-regulated genes suggest a significant effect of subjective circadian time of harvesting on expression levels in all three mutant genotypes. Significance of differential gene expression in *xct* mutants assessed using the Bioconductor package edgeR (Robinson et al., 2010), false discovery rate < 0.05, with no fold-change threshold imposed.

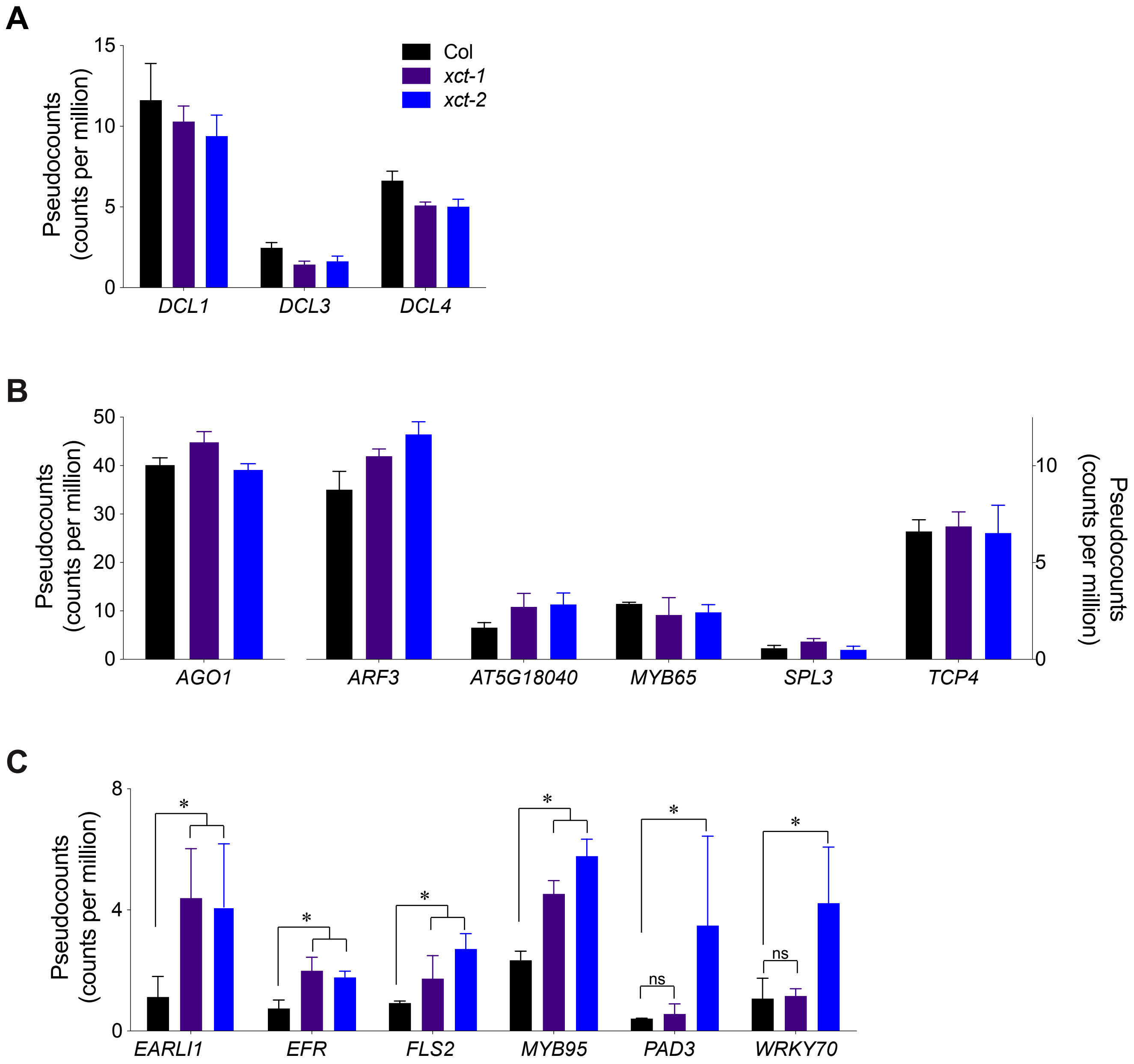

Supplementary Figure 2. Expression of small RNA- and immune-related genes in *xct* mutants. Expression levels of the indicated genes in wild-type Col and the *xct* mutants as determined by RNA-seq. Pseudocounts correspond to the average numbers of reads mapped to the indicated loci in each genotype, normalized by library size. (A) Genes involved in processing small RNAs are not significantly differentially expressed in *xct* mutants. (B) Expression levels of genes negatively regulated by small RNAs are not significantly differentially expressed in *xct* mutants. (C) Expression levels of pathogenesis-related genes are statistically significantly elevated in both *xct-1* and *xct-2* (*EARL1, EFR, FLS2, MYB95*) or in *xct-2* but not *xct-1* mutants (*PAD3, WRKY70*) mutants. Significance assessed using the Bioconductor package edgeR (Robinson et al., 2010), false discovery rate < 0.05.

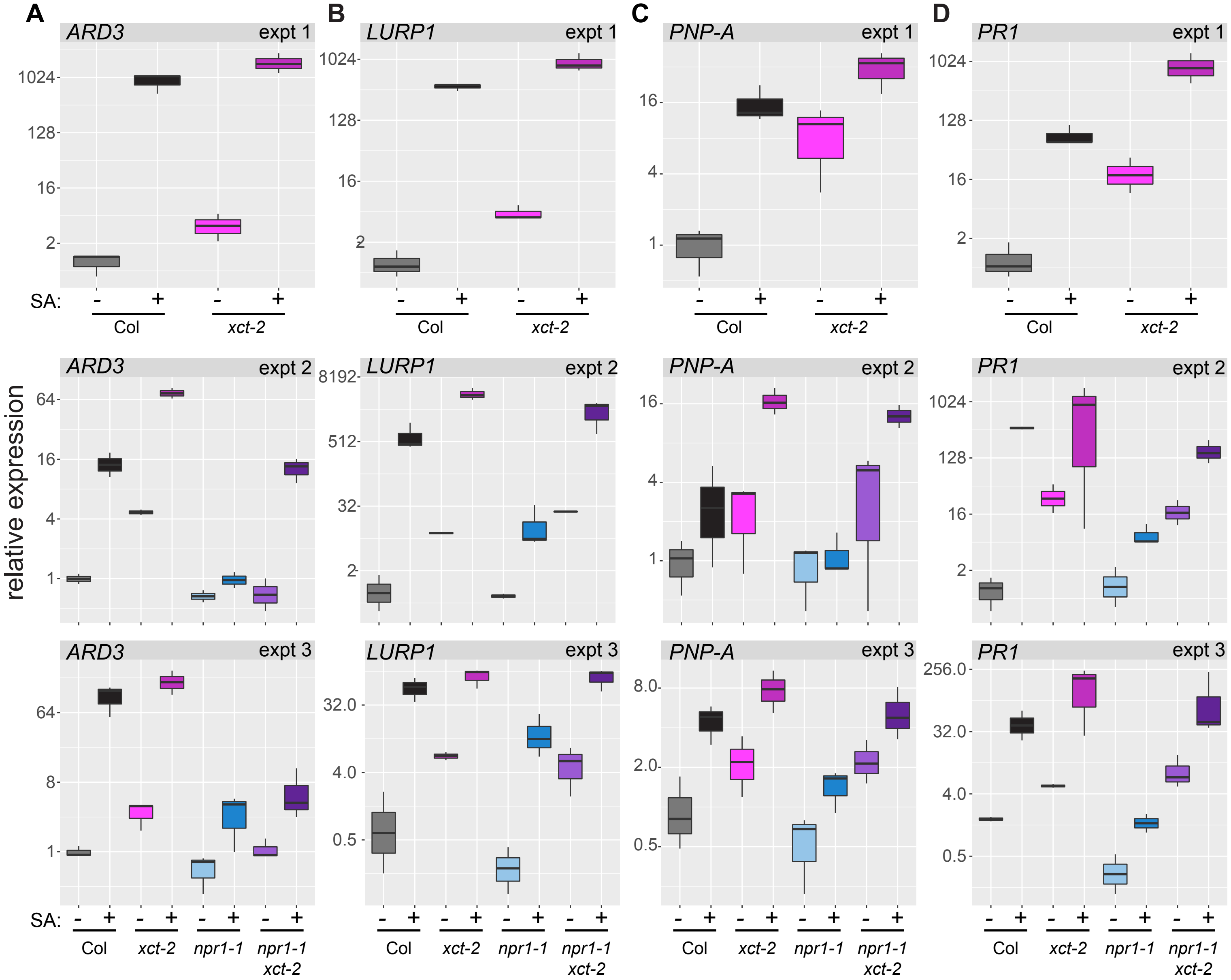

Supplementary Figure 3. Loss of XCT function increases salicylic acid responsiveness. Plants of the indicated genotypes were either mock-treated or sprayed with 0.3 mM salicylic acid and samples were collected 24 hours later in three independent trials. Expression levels of the indicated genes were determined by qRT-PCR and normalized to *PP2C*. Using linear mixed-effect models with treatment and genotype as fixed effects and trial as a random effect, we found a significant effect of the *xct-2* genotype on responsiveness to SA for the *PR1* ($p = 0.02$), *LURP1* ($p = 0.01$), and *PNP-A* ($p =0.02$) genes. Lines within boxes represent medians; upper and lower margins of the boxes correspond to the first and third quartiles; whiskers extend to 1.5 times the interquartile range. Note the log2 scale on the y-axes.

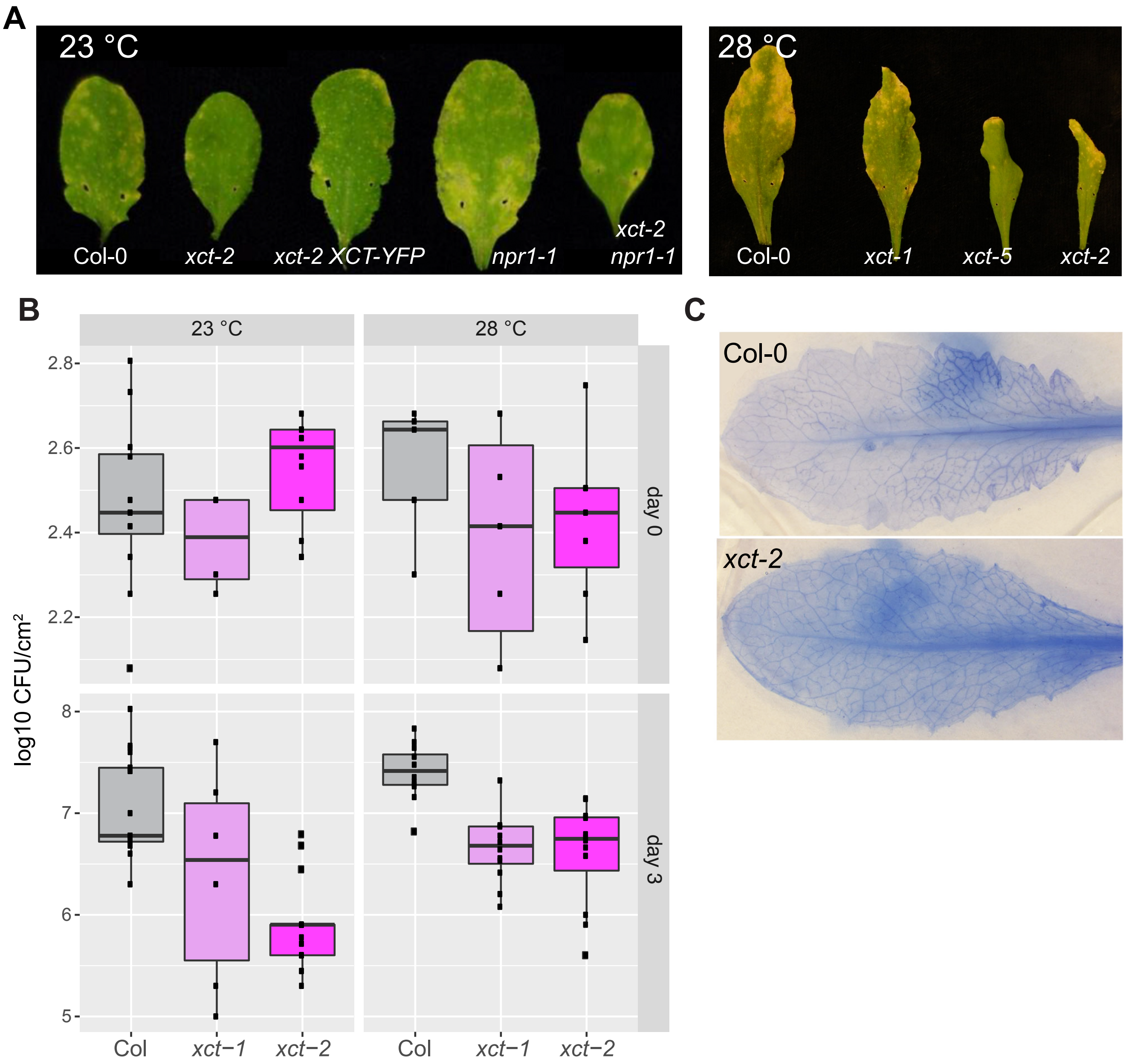

Supplementary Figure 4. Enhanced immune responses in *xct* mutants are independent of temperature. (A) The indicated genotypes were either grown at 23 °C or 28 °C and inoculated with *Pseudomonas syringae* pv. *tomato* DC3000. Images were taken three days after inoculation. (B) Data from Figure 4 are replotted here. Using linear mixed-effect models with temperature as a fixed effect and trial as a random effect, we found a significant effect of temperature on the number of bacterial colonies on day 3 ($p = 2\ e^{-4}$) but not on day 0 ($p = 0.5$). (C) Trypan blue staining of leaves of Col-0 and *xct-2* plants grown at 22 °C did not reveal spontaneous cell death in either genotype.

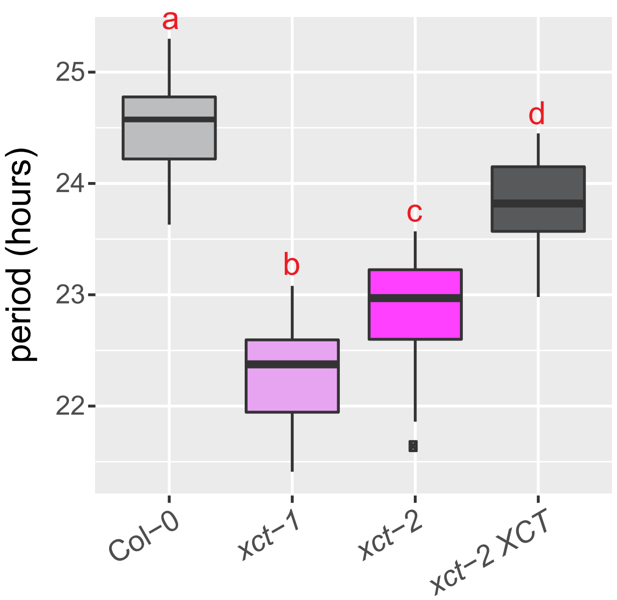

Supplementary Figure 5. Circadian period phenotypes of *xct* alleles. Period of *CCR2::LUC+* activity of the indicated genotypes. Seedlings were grown in light/dark cycles (12 hr light/12 hr dark) for 6 days and then released to constant red plus blue light for luciferase imaging. Box edges represent the 75th and 25th percentiles while the mid-line in each box represents the medians and the whiskers reprsent the largest or smallest values within 1.5 times the interquartile range. Letters represent treatments with significantly different means (P <0.0005; one-way ANOVA with multiple pairwise comparisons carried out using Tukey's HSD).

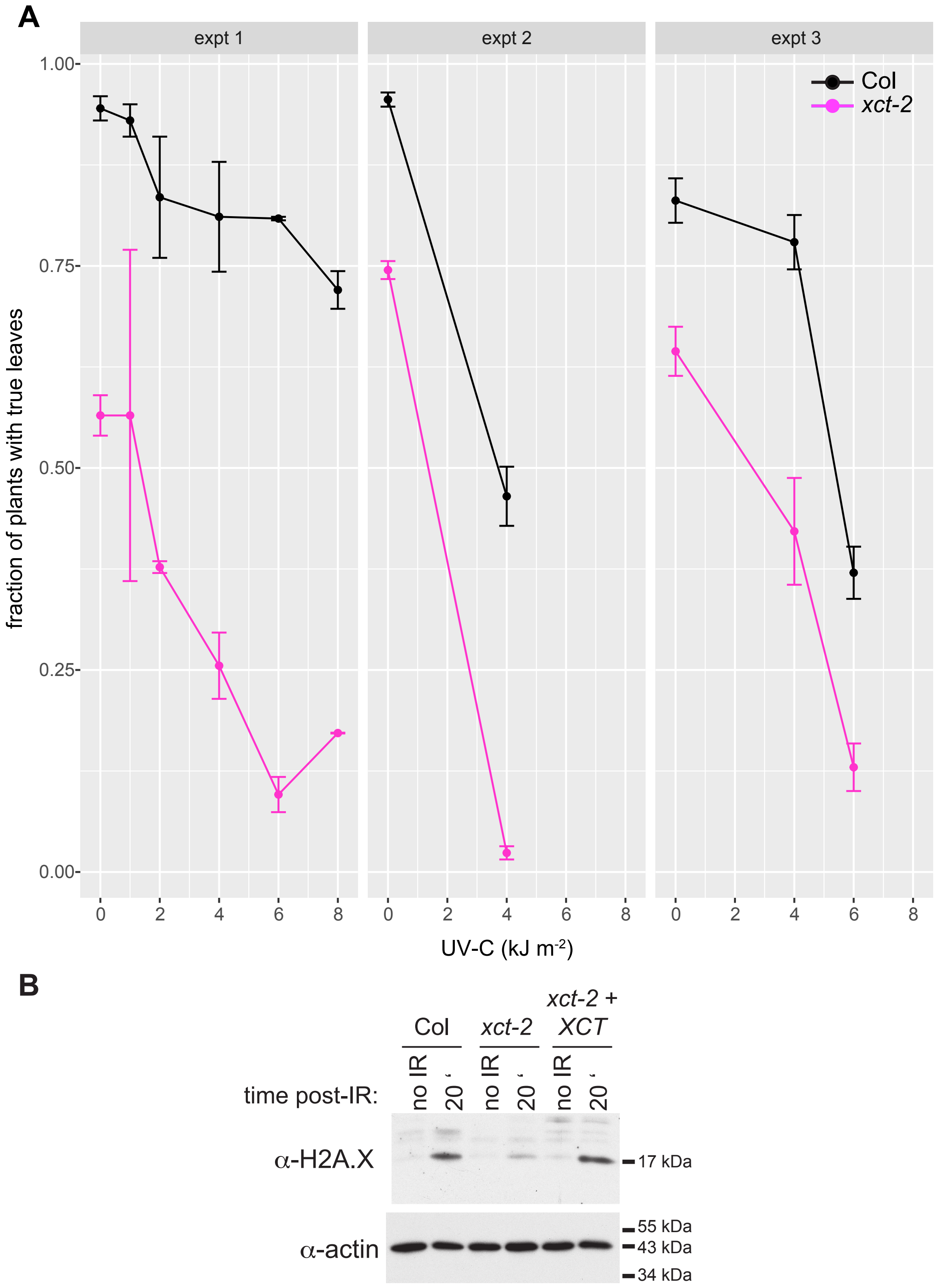

Supplementary Figure 6. ***xct-2*** **mutants are deficient in response to DNA damaging agents.** (A) Formation of true leaves was determined for *xct-2* and wild-type Col seven days after exposure to the indicated fluences of UV-C irradiation as described in the legend for Figure 6. Fractions of plants with true leaves differ from those plotted in Figure 6 because a different investigator scored each set of experiments.Error bars represent SEM. Using a linear mixed effect model with genotype and irradiance as fixed effects and trial as random effect, the genotype by irradiance interaction was found to be significant at $p = 0.005$. (B) Levels of histone H2A.X phosphorylation after γ irradiation were determined as described in the legend for Figure 6.
